# Antioxidative, cytoprotective and whitening activities of fragrant pear fruits at different growth stages

**DOI:** 10.3389/fnut.2022.1020855

**Published:** 2022-09-30

**Authors:** Hui Jiang, Fei Wu, Xi Jiang, Yun-Feng Pu, Li-Rong Shen, Cui-Yun Wu, Hong-Jin Bai

**Affiliations:** ^1^Engineering Laboratory of Chemical Resources Utilization in South Xinjiang of Xinjiang Production and Construction Corps, Tarim University, Alar, China; ^2^College of Life Sciences, Tarim University, Alar, China; ^3^The National and Local Joint Engineering Laboratory of High Efficiency and Superior-Quality Cultivation and Fruit Deep Processing Technology of Characteristic Fruit Trees in South Xinjiang, Tarim University, Alar, China; ^4^College of Food Science and Engineering, Tarim University, Alar, China; ^5^College of Biosystems Engineering and Food Science, Zhejiang University, Hangzhou, China

**Keywords:** fragrant pear, phenolic compounds, antioxidant activity, cytoprotective activity, whitening activities

## Abstract

Pear is one of the most popular fruits in the world. With the fruit ripening, a series of physiological changes have taken place in fragrant pear, but up to now, the research on the metabolism and biological activity of phenolic compounds in different growth stages of fragrant pear is still lacking. In this study, four kinds of Xinjiang pears were selected as research objects, and the changes of phenolic content, antioxidant capacity, cell protection and whitening activity during fruit development were analyzed. The results showed that the phenolic content and antioxidant capacity of four pear varieties presented a decreasing trend throughout the developmental stages. The phenolic content and antioxidant activity of the four pears in the young fruit stage were the highest, and the active ingredients of the Nanguo pear were higher than the other three pear fruits. Pear extract could protect cells by eliminating excessive ROS in cells, especially in young fruit stage. The western blot results showed that the extract of fragrant pear in the young fruit stage could inhibit the expression of TYR, TYR1 and MITF in B16 cells, and it was speculated that the extract of fragrant pear in the young fruit stage might have good whitening activity. Therefore, the findings suggest that young pear display a good antioxidant potential and could have a good application prospect in food preservation and health product industry.

## Introduction

In recent years, with the continuous development of the social economy and the continuous improvement of people’s quality of life, antioxidant and whitening have become two major focuses of attention. Under normal circumstances, the production and clearance of free radicals in cells are in a dynamic balance. And free radicals have various physiological functions such as maintaining cell function, eliminating toxins in the body and transmitting signals (1). When the body is stimulated by external stressors, excessive free radicals will be produced. At this time, macromolecules in the body are decomposed and a large number of harmful products are produced, which leads to lesions in normal cells and tissues of the body, and promotes the pathological development of diseases such as inflammation, cancer and diabetes (2–5). Previous studies showed that reactive oxygen species (ROS) were the main culprit in the oxidation and aging of the body (6). ROS with strong oxidative activity would be produced in biochemical reaction. Excessive ROS could induce oxidative stress, resulting in oxidative damage and senescence of cells (7). Therefore, inhibiting the production of excessive ROS would be of great significance for preventing cellular oxidation and aging. In recent years, the research and development of antioxidant drugs has also emerged. Studies have shown that active ingredients such as flavonoids, polyphenols, polysaccharides, and alkaloids in traditional Chinese medicine or natural medicines show good antioxidant activity (8). Antioxidants are also one of the main mechanisms of skin whitening (9). Melanin is a high molecular biological pigment synthesized by melanocytes, which is distributed in the dermis of the skin and determines the color of the skin. The appropriate melanin is beneficial to skin health and can effectively absorb ultraviolet rays, thereby protecting the skin from photoaging. However, excessive accumulation of melanin will not only make the skin black, but also lead to freckles, vitiligo and even skin cancer (10, 11). The production of melanin is mainly regulated by tyrosinase (TYR), which is the key rate-limiting enzyme in the initiation reaction of melanin (12). The production rate and yield of melanin are also regulated by tyrosinase-related protein (TRP-1 and TRP-2) and mi-crophthalmia associated transcription factor (MITF) (13). The skin color of the human body is mainly determined by the content and distribution of melanin in the skin, and tyrosinase is the rate-limiting enzyme in the melanin synthesis pathway. By inhibiting its activity, the skin whitening effect can be achieved. To this end, tyrosinase is also the main target for the screening of active ingredients in whitening and freckle-removing cosmetics. In the process of catalyzing melanin synthesis, the presence of oxygen is a necessary condition. Therefore, the antioxidant effect plays an important role in reducing the synthesis of melanin. It may reduce melanin synthesis by blocking or attenuating tyrosinase activity (14). Studies have found that Chinese herbal medicines and chemical components in plants can effectively inhibit the production of melanin. Because plant extracts are natural, mild, and less irritating, they are favored by consumers. Therefore, it has become a research hotspot in the cosmetics industry to find a relatively safe and effective cosmetic additive ingredient from natural plants that has no side effects on the human body (15).

Pears are the third largest consumed fruit next to apples and citrus in China. Pears contain fat, protein, crude fiber, sugar, calcium, iron, phosphorus, vitamins and polyphenols, which have the effect of nourishing yin, clearing heat and lowering blood pressure (16). Nanguo pear, *Pyrus ussuriensis Maximis* is one of the best varieties in the autumn pear system with the advantages of strong cold resistance, disease resistance, high juice content and good fresh eating quality (17). Aksu Juju pear and Luntai Juju pear belong to Xinjiang pear system with the characteristics of high yield, high quality and strong cold resistance. Hong xiang pear is one full of red pear varieties, a current largest and reddest new variety of intense red bud variation with strong tree, excellent quality and adaptability characteristics. The pear fruit with characters of fragrant, juicy and nutritious is welcome by consumers. Phenolic substances including phenolic acids and flavonoids, such as arbutin, chlorogenic acid, catechin and caffeic acid is the main chemical composition with antioxidant, free radical scavenging and antibacterial effects in pear fruit (18). Pear phenolic compounds are a pure natural antioxidant extracted from pears, and its main component is flavonoids. In recent years, a large number of studies have shown that pear phenolic compounds have various pharmacological functions, such as antibacterial, antioxidant, anti-arteriosclerosis, and hair growth. These pear fruits are sweet and juicy, fragrant and refreshing, crispy and less residue, and rich in phenolic substances with strong antioxidant activity, such as chlorogenic acid, rutin, epicatechin, catechin, etc. consumer favorite (19). The antioxidative and anti-melanin deposition effects of fragrant pear provide a good pharmacodynamic basis for the research and development of antioxidative and whitening cosmetic products.

In this study, the total phenols and flavonoids in the fruits of Nanguo Pear, Aksu Juju Pear, Hongxiang Pear and Luntai Juju Pear at different growth stages were studied. The ability of scavenging oxygen free radicals *in vitro* during pear fruit development was explored, and HEK-293 cell damage was induced by oxidative stressors to establish a cell oxidative damage model. The effects of fragrant pear extract on the survival rate and ROS level of HEK-293 cells induced by oxidative stressors were determined by MTT method and cell flow analyzer, respectively, and the effect of fragrant pear extracts in different periods on oxidative stress-induced HEK-293 cells was evaluated in turn. In addition, in this study, the mouse B16 cells were used as the research object, and the cell model with high melanin expression induced by α-MSH was constructed to study the inhibitory effect of fragrant pear extract on α-MSH-induced melanin production in B16 cells and preliminarily explore its mechanism, in order to provide scientific basis for the application of fragrant pear extract in cosmetics or pharmaceuticals.

## Materials and methods

### Plant materials

**FIGURE 1:**
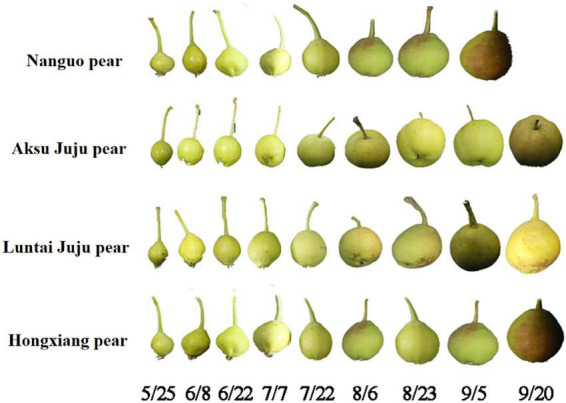
Fruit at the developmental stage of the Nanguo pear, Aksu Juju pear, Hongxiang pear and Luntai Juju pear.

Four different varieties of Xinjiang pears, namely, Nanguo pear, Aksu Juju pear, Hongxiang pear and Luntai Juju pear, were picked from Xinjiang Tarim University as the experimental material for this study. Each variety were selected from 3 trees of uniformity, good growth of the plant, since May 25 (50 days after flowering) began sampling, every 15 days regular sampling, each time in the east, west, north, south and central 5 directions randomly taken, each tree selected uniform size, no disease or pest 10 fruit, a total of 30 fruit, until the pear fruit food fully mature. The Aksu Juju pear, the Hongxiang pear and the Luntai Juju pear are all picked until 20 September and the Nanguo pear until 5 September (Figure 1). Immediately after picking, the pears were placed in a cooler with ice packs and taken back to the laboratory.

HEK-293 cells and B16 cells were purchased from the Chinese Academy of Sciences. The MEM, DMEM media and 2′, 7′-dichlorofluorescein diacetate (DCFH-DA) were purchased from Beijing Solarbio Biotechnology Co., Ltd. (Beijing, China); Arbutin, catechin, chlorogenic acid, epicatechin, rutin were purchased from Aladdin Reagents Ltd (Shanghai, China); Sodium bicarbonate, sodium carbonate, etc. were purchased from Tianjin Zhiyuan Chemical Reagent Co.; 2N Folin-Ciocalteu reagent were purchased from Shanghai Lida Biotechnology Co.; Methanol, sodium nitrite, aluminum nitrate, sodium hydroxide, 2.6-dichlorophenol indophenol, ascorbic acid were purchased from Shanghai Yuanye Biotechnology Co., Ltd. (Shanghai, China).

### Extraction of phenols

A certain amount of lyophilized powder was weighed, ultrasonically extracted with 80% methanol at room temperature for 30 min, centrifuged to obtain a supernatant, and stored at –20°C for subsequent use. The extracts were also used in subsequent experiments. The assays were all referenced to the method of Pu et al. (20).

### Total phenolic content

The total phenol content was determined by the Folin-Ciocalteu colorimetric method. 0.2 mL of the extract was taken up, 10 mL of distilled water and 2 mol/L of Folin-Ciocalteu reagent were added, mixed and allowed to stand for 5 min, 1.0 mL of 15% Na_2_CO_3_ solution was added, mixed and the reaction was carried out for 2 h at room temperature and protected from light. The absorbance values of the samples were determined by UV-2600 spectrophotometer (Shimadzu Instruments Ltd., Jiangsu, China). After scanning the full-wavelength absorption spectrum of the sample, 746 nm, the maximum absorption, was selected as the measurement wavelength. And gallic acid was used as standard. The results were repeated three times and expressed as mg GAE/g DW.

### Total flavonoids content

The total flavonoid content (TFC) was determined by the aluminum nitrate color development method. 5 mL of the extract was taken up with 0.5 mL of 5% NaNO_3_ solution, added to a 15 mL centrifuge tube, shaken well and reacted at room temperature for 6 min. 0.5 mL of 10% Al(NO_3_)_3_ solution was added, mixed well and reacted at room temperature for 6 min. 4 mL of 4% NaOH solution was added and reacted at room temperature for 20 min. The absorbance values of the samples were measured at 502 nm and repeated three times. The results were expressed as mg RE/g DW using rutin as the standard.

### Determination of phenol single component content by high performance liquid chromatography

The extract was filtered through a 0.45 μm microfiltration membrane into a 1.0 mL injection vial and the phenolics were determined using LC-20A liquid chromatography with RID20A detector (Shimadzu Corporation, Japan). The column was a Spursil C18 (250 × 4.6 mm, i.d.) column (Dikma Ltd., Tianjin, China), and the column was maintained at 30°C. UV absorption of the high performance liquid chromatography (HPLC) eluates was recorded at wavelengths of 280 nm and 360 nm for real-time monitoring of the peak intensity. The mobile phase was 0.11 mol/L formic acid (eluant B) and methanol (eluant A), and the flow rate was 0.7 mL/min and the injection volume was 10 μL. The gradient program of samples were as follows: 0–5 min, 90–85% of B, 5–6 min, 85–80% of B, 6–10 min, 80% of B, 10–11 min, 80–75% of B, 11–15 min, 75–60% of B, 15–25 min, 60% of B, 25–38 min, 60–41% of B, 38–48 min, 38–37% of B, 48–49 min, 37–34% of B, 49–59 min, 34–33% of B, 59–63 min, 0 of B, 63–73 min, 0 of B, 73–74 min, 0–90% of B, 74–85 min, 90% of B. Each sample was repeated three times and the phenolic content of the samples was expressed as μg/g DW. The determination conditions and method were based on the method of Pu et al. (20).

### Determination of antioxidant activity

Aspirate 20 μL of sample solution into a 96-well microplate, add 200 μL of DPPH solution, shake at 300 r/min for 30 min and scan the absorbance at 517 nm using an enzyme marker. Trolox was used as a standard and the results were expressed as μmol TE/g DW.

Aspirate 20 μL of sample solution into a 96-well microtiter plate, add 200 μL ABTS solution, shake at 300 r/min for 10 min, then scan the absorbance value at 734 nm and use Trolox as a standard, the results are expressed as μmol TE/g DW.

Aspirate 20 μL of sample solution into a 96-well microplate, add 200 μL of FRAP solution, shake at 300 r/min for 10 min and scan the absorbance at 593 nm using an enzyme marker. Trolox was used as a standard and the results were expressed as μmol TE/g DW.

### Measurement of intracellular reactive oxygen species

The effect of pear extracts on intracellular ROS production were evaluated by a ROS assay kit (21). HEK-293 cells were cultured in MEM medium supplemented with 10% fetal bovine serum (FBS), 100 U/mL penicillin and 100 μg/mL streptomycin. The cell suspension was seeded in a 6-well plate (0.8 mL, 1.0 × 10^6^ CFU/mL) and treated with 200 mg/mL the extract of Nanguo pear, Aksu Juju pear, Hongxiang pear and Luntai Juju pear for 6 h. after 0.5 h of incubation with TBHP, the cells were stained by DCFH-DA (2 μM) for 0.5 h. The ROS levels in HEK-293 cell were determined and analyzed using flow cytometry (Becton, Dickinson and Company, New Jersey, USA) with excitation wavelength at 489 nm and emission wavelength at 524 nm, and 10,000 events were collected.

### Measurement of cytoprotective activity

The cytoprotective activity of pear extracts was determined using the MTT method as described previously (22). HEK-293 cells were cultured in MEM medium supplemented with 10% FBS, 100 U/mL penicillin and 100 μg/mL streptomycin. The cell suspension was seeded in a 96-well plate (1 × 10^6^/well), incubated at 37°C for 12 h, after adding the pear extracts (200 mg/mL) for 0.5 h, adding H_2_O_2_ solution (final concentration 700 μM) for 6 h, and then adding 20 μL MTT stock solution (5 mg/mL) to each well, and then the cells were further incubated for 4 h. Finally, remove the medium and add DMSO (200 μL) to each well to dissolve the formazan. After 10 min, the absorbance was measured at 570 nm by PT-3502C full wavelength ELISA instrument (Beijing Putian Xinqiao Technology Company Limited, Beijing, China).

### Measurement of whitening-related protein levels

Effects of pear extract on the expression of melanin production-related proteins were evaluated as described previously (21). B16 cells were cultured in DMEM medium supplemented with 15% FBS, 100 U/mL penicillin and 100 μg/mL streptomycin. The cell suspension was seeded in a 6-well plate (1 × 10^6^/well), incubated at 37°C for 12 h, and pre-treated with 0.5 μM α-MSH or pear extract (200 mg/mL), and after 48 h, the cells were lysed and total cell proteins were extracted. The protein concentration was determined by Bradford method, and 30 μg of protein was added to each electrophoresis channel for polyacrylamide gel electrophoresis, and semi-dry transfer to PVDF membrane. The primary antibodies (1:1,000) of TYR, TRP-1, MITF, and GAPDH were added for immunoblotting, and then the corresponding secondary antibodies diluted 1:5,000 were used for hybridization. Using ELC reaction, dark room development, exposure. Bio-Rad software analyzed the protein content of each electrophoresis band, and the result was expressed as the ratio of the gray value of each electrophoresis band to the gray value of GAPDH.

### Statistical analysis

Data were statistically analyzed using Origin 2018 software and results were expressed as mean ± standard deviation for one-way ANOVA; SPSS 25 statistical software was used for significance analysis and Duncan’s new complex polar difference method was used for multiple comparisons.

## Results and discussion

### Changes of total phenols and total flavonoids in pear fruit during growth

**FIGURE 2:**
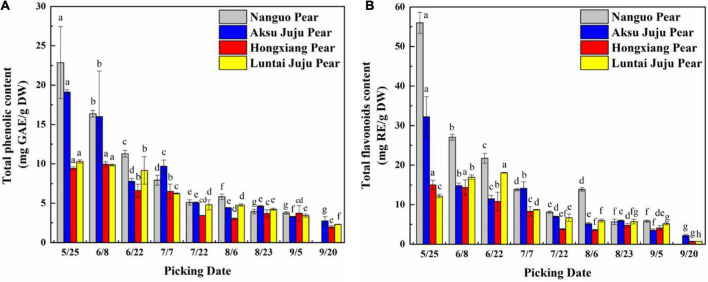
Changes in the TPC and TFC in pear fruits. **(A)** Total phenol content; **(B)** total flavonoid content (mean ± SD, *n* = 3). Bars with different letters differ significantly (*p* < 0.05).

The Total phenolic contents (TPC) in fruits of four pear varieties showed a decreasing trend during development (Figure 2A). TPC at 5/25 were relatively high, and the differences among varieties were significant (*p* < 0.05). TPC tended to be highest in Nanguo pear (22.86 mg GAE/g DW), followed by the Aksu Juju pear (19.11 mg GAE/g DW), and the Hongxiang pear was the lowest (9.43 mg GAE/g DW). TPC of the Nanguo pear drops to a minimum (3.77 mg GAE/g DW) at 9/5, TPC of the Aksu Juju pear, Hongxiang pear and Luntai Juju pear drops to a minimum (2.74 mg GAE/g DW, 1.98 mg GAE/g DW and 2.31 mg GAE/g DW) at 9/20.

The trends in TFC of the Nanguo pear, Aksu Juju pear and Hongxiang pear were consistent (Figure 2B) during development. The TFC of Nanguo pear was the highest at 5/25 (55.98 mg RE/g DW), followed by the Aksu Juju pear (32.22 mg RE/g DW). TFC of Luntai Juju pear showed a trend of slowly increasing and then decreasing, with the highest content at 6/22 (18.09 mg RE/g DW). With the development of the fruit, the TFC of Nanguo pear decreased to the lowest level at 9/5 (5.85 mg RE/g DW), the content of Aksu Juju pear, Hongxiang pear and Luntai Juju pear decreased to the lowest level at 9/20 (2.21 mg RE/g DW, 0.69 mg RE/g DW and 0.75 mg RE/g DW), respectively. And TPC and TFC of the four kinds of pear fruits studied in this paper were: Nanguo pear > Aksu Juju pear > Luntai Juju pear > Hongxiang pear. The effect of ripening stages on TPC and TFC was significant. This phenomenon may be caused by some enzymes that are involved in the synthesis of phenolic compounds. For example, the coupling of 1,2-rhamnosyltransferase and flavanone-7-O-glucosides accelerates the biosynthesis of flavonoids in the early growth periods, whereas an insufficient supply of flavonoid aglycone might explain the decrease in flavonoids during the ripening stage of fruits (23).

In summary, TPC and TFC of pear fruit is really high in the young fruit stage, which was also found in a study by Cho et al. (24) on the dynamics of the phenolic content of seven pear varieties. In nature, the color of fruit usually changes with the content of phenolic compounds, such as apples, pears and blueberry in which anthocyanin or flavonoid pigments accumulate expressing ripening of fruit (25). Excessive phenolic compounds in young fruits can effectively prevent animals from damaging the seeds due to the bitter taste. At the ripening stage, sugars and phenolic compounds are accumulated, fruits are softer, darker, sweeter and more attractive for seed dispersers (26).

### Composition and content of phenolics in pear fruit during growth

**FIGURE 3:**
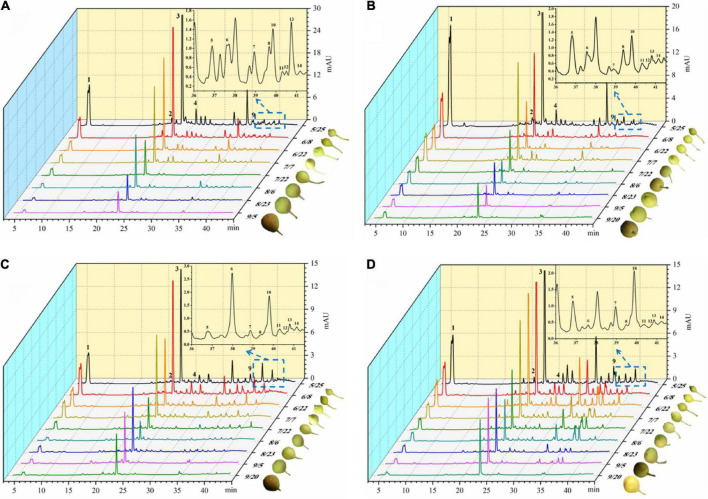
Dynamics of four pear high performance liquid chromatography (HPLC). **(A)** Liquid chromatogram of Nanguo pear; **(B)** liquid chromatogram of Aksu Juju pear; **(C)** liquid chromatogram of Hongxiang pear; **(D)** liquid chromatogram of Luntai Juju pear. 1 Arbutin, 2 Catechin, 3 Chlorogenic acid, 4 Epicatechin, 5 Kaempferol hexoside-dideoxyhexoside, 6 Rutin, 7 Kaempferol-3-O-rutinoside, 8 Quercitrin, 9 Quercetin rhamnoside, 10 Isorhamnetin-3-O-rutinoside, 11 Kaempferol-3-O-glucoside, 12 Isorhamnetin-3-O-glucoside, 13 Kaempferol-3- acetylglucoside, 14 Isorhamnetin-3-acetylhexoside.

**FIGURE 4:**
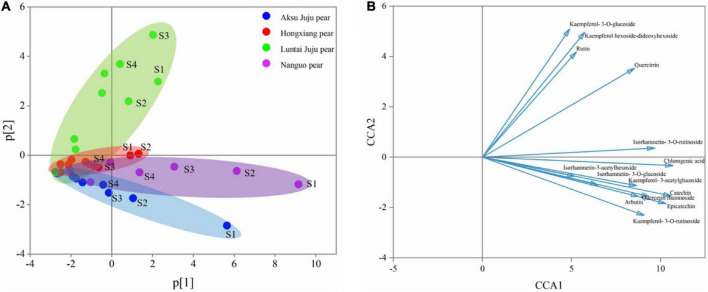
PCA principal component analysis of phenolic compounds from four pear species. **(A)** PCA score plot and **(B)** PCA loading plot of all measured primary phenolic compounds profiles. S1, S2, S3, and S4 correspond to 5/25, 6/8, 6/22, and 7/7, respectively, same below.

**FIGURE 5:**
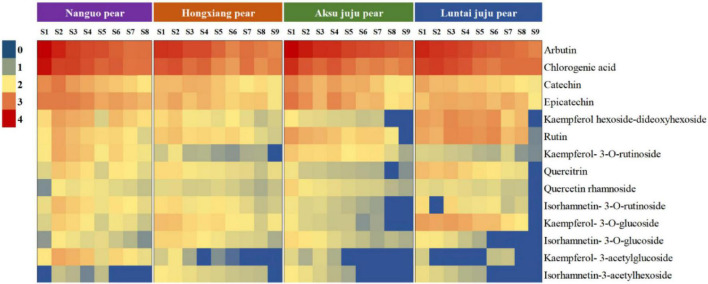
Thermogram of four pear phenolic compounds.

With the development of the fruit, Arbutin, Catechin, Chlorogenic acid, Epicatechin, Kaempferol hexoside-dideoxyhexoside, Rutin, Kaempferol-3-*O*-rutino side, Quercitrin, Quercetin rhamnoside, Isorhamnetin-3-*O*-rutinoside, Kaempferol-3-*O*-glucoside, Isorhamnetin-3-*O*-glucoside, Kaempferol-3- acetylglucoside, Isorhamne tin-3-acetylhexoside were detected in the four pear fruit species (Figure 3), the 14 phenolic compounds were highest in the young fruit growing stage (5/25–6/22), among which arbutin and chlorogenic acid were the main components of pear fruit phenolics, and arbutin was the highest, in line with the trend for total phenols and total flavonoids. Wang et al. (19) also found the highest levels of arbutin and chlorogenic acid in a study of 10 pear varieties, and Zhang and Liu (27) used Yali as samples and also demonstrated that arbutin and chlorogenic acid are the main phenolic substances in pear fruit. In the young fruit stage, the highest arbutin and chlorogenic acid content was found in the Nanguo pear and the Aksu Juju pear, which were 14104.22 μg/g DW, 22403.53 μg/g DW and 10614.57 μg/g DW, 6761.10 μg/g DW, followed by 8,281.87 μg/g DW and 3,816.91 μg/g DW for the Luntai Juju pear, and the lowest 5,663.91 μg/g DW and 2013.04 μg/g DW for the Hongxiang pear. Nanguo pear, Aksu Juju pear, Hongxiang pear and Luntai Juju pear at the young stage were 12.51 times, 15.10 times, 9.89 times, 12.88 times and 10.23 times, 8.84 times, 2.87 times and 3.48 times higher than the ripe stage, respectively. As can be seen from Figure 4A, different pear varieties have obvious differences between the phenolic compounds, and the same pear varieties in the development of the early content of significant differences, no significant changes in the late development, analysis of the reasons may be related to the early development of the fruit phenolic synthesis is relatively rapid, and the late gradually become slow or stop, while the transformation of phenolic substances accelerated, thus causing the total amount of decline. As can be seen from Figures 3, 4B, ten phenolic compounds, including Arbutin, Catechin, Chlorogenic acid, Epicatechin, Kaempferol-3-*O*-rutinoside, Quercetin rhamnoside, Isorhamnetin-3-*O*-rutinoside, Isorhamnetin-3-*O*-glucoside, Kaempferol-3-acetylglucoside and Isorhamnetin-3-acetylhexoside, showed a significant downward trend with fruit ripening. Four other phenolic compounds, including Kaempferol hexoside-dideoxyhexoside, Rutin, Kaempferol-3-O-glucoside and Quercitrin, showed an increasing and then decreasing trend. As can be seen from Figure 5, arbutin having the highest content, followed by chlorogenic acid, and Isorhamnetin-3-acetylhexoside having the lowest content, and the content of phenolic compounds in Nanguo pear and Aksu Juju pear was relatively high at the young fruit stage, with more obvious changes in content throughout the growth and development period. Currently, synthetic antioxidants are widely used in cosmetics and health care products, but long-term use will be accompanied by certain toxic side effects (27). Therefore, the search for natural antioxidants has become a popular field, and the extracts of Nanguo pear and Aksu Juju pear found in this study can be used as high-quality natural extraction resources of chlorogenic acid and arbutin.

### Changes in antioxidant capacity of pear fruit during growth

**FIGURE 6:**
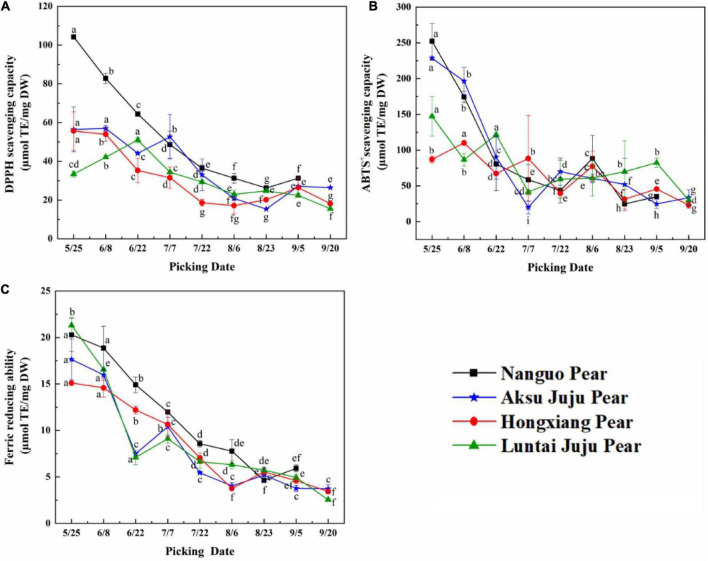
Changes in the scavenging ability of DPPH, ABTS^+^ free radicals and FRAP of pear fruits. **(A)** DPPH free radical scavenging ability; **(B)** ABTS^+^free radical scavenging ability; **(C)** ferric reducing ability (mean ± SD, *n* = 3). Bars with different letters differ significantly (*p* < 0.05).

As can be seen from Figure 6, The phenolic antioxidant indexes of Nanguo pear, Aksu Juju pear, Hongxiang pear and Luntai Juju pear showed a decreasing trend throughout the growth and development period, although with slight fluctuations during the period.

When evaluating antioxidant capacity in terms of DPPH free radical scavenging capacity (Figure 6A), the Luntai Juju pear was strongest on 6/22 (51.04 ± 1.44 μmol TE/g DW), Aksu Juju pear was strongest in capacity on 6/8 (56.97 ± 1.39 μmol TE/g DW) and the other two pear species were strongest at 5/25 with 104.18 ± 0.72 μmol TE/g DW and 55.66 ± 9.91 μmol TE/g DW, respectively. The four pear species had 3.97, 3.70, 2.76, and 3.30 times higher scavenging capacity for DPPH free radicals in young fruit than in mature fruit.

For ABTS^+^ free radical scavenging capacity (Figure 6B), Hongxiang pear was strongest at 6/8 (110.20 ± 3.35 μmol TE/g DW), the other three pear species had the highest capacity at 5/25 with 252.49 ± 24.84 μmol TE/g DW, 228.58 ± 2.79 μmol TE/g DW and 147.31 ± 27.72 μmol TE/g DW, respectively. The four pear species had 10.23, 9.22, 4.74, and 4.89 times higher scavenging capacity for ABTS^+^ free radicals in young fruit than in mature fruit.

As can be seen from Figure 6C, the four pears had the strongest FRAP values at 5/25 with 20.29 ± 1.79 μmol TE/g DW, 17.64 ± 2.18 μmol TE/g DW, 15.10 ± 0.10 μmol TE/g DW and 21.31 ± 0.81 μmol TE/g DW, respectively. The FRAP values of the four pear species had 3.43, 4.73, 4.40, and 8.35 times higher in young fruit than in mature fruit.

In summary, although the three indicators for evaluating antioxidant capacity differed in terms of trends or antioxidant strength, the antioxidant capacity of all four types of pear fruit showed the highest antioxidant capacity at the young fruit stage, consistent with the higher total phenol and TFC at the young fruit stage. The same trend was observed in bayberry fruit development (28). Interestingly, the antioxidant activity values of the samples from the same growth month were different probably because of the various mechanism of the three methods (29). The three antioxidant capacity is from strong to weak: Nanguo pear > Aksu Juju pear > Luntai Juju pear > Hongxiang pear. This result is basically consistent with that of phenolic compound content.

### Study on scavenging reactive oxygen species of fragrant pears in different periods

**FIGURE 7:**
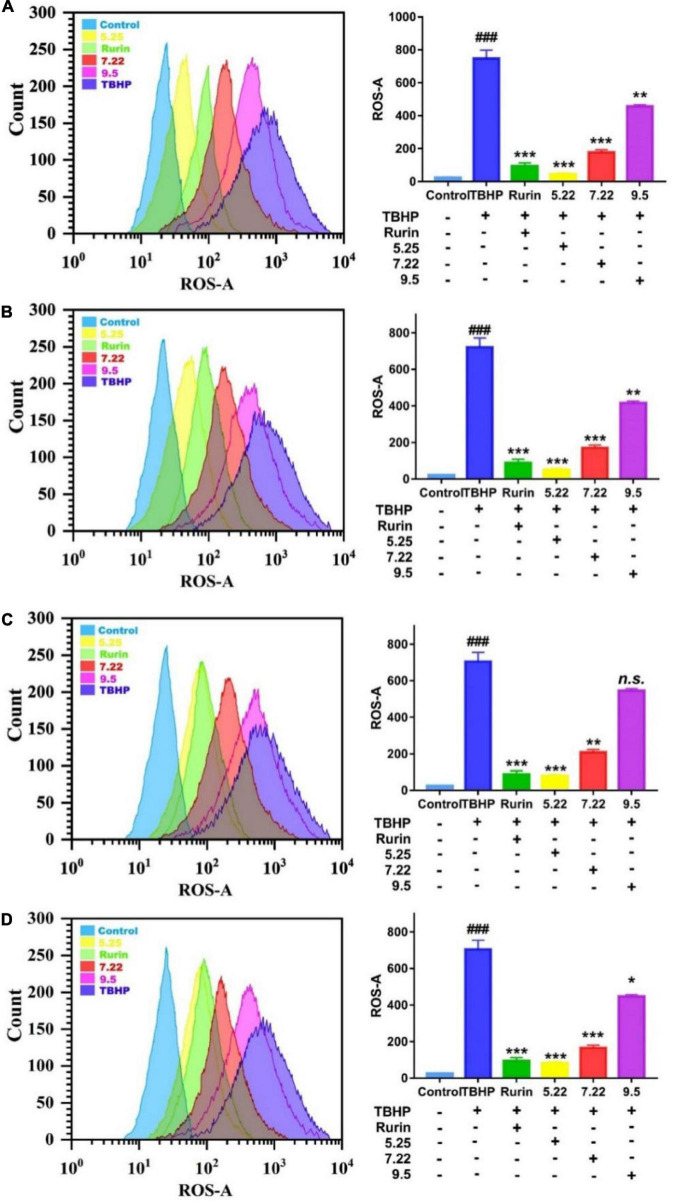
Effect of nanguo pear **(A)**, Aksu Juju pear **(B)**, Hongxiang pear **(C)** and Luntai Juju pear **(D)** on ROS generation in TBHP-stimulated HEK-293 cell. Cells (0.8 mL, 1.0 × 10^6^ CFU/mL) were treated with 200 mg/mL the extract of Nanguo pear, Aksu Juju pear, Hongxiang pear and Luntai Juju pear for 6 h, after 0.5 h of incubation with TBHP, were stained by DCFH-DA. The ROS levels in HEK-293 cell were determined and analyzed using flow cytometry. All data represent means ± SD of three independent experiments. ^###^*p* < 0.001, compared with control group, ***p < 0.001, **p < 0.01 and *p < 0.05 related to TBHP alone.

Under normal physiological conditions, the production and scavenging of ROS in the body are in a dynamic balance, and an appropriate amount of ROS helps to improve the immunity of the body and the phagocytic ability of macrophages (30). However, when the body is stimulated by the outside world, the balance of ROS production and scavenging is broken, and a large amount of ROS will accumulate in the body. Excessive ROS will cause oxidative damage to cell membranes, lipids, proteins, and DNA, and eventually lead to cell apoptosis (31). In this study, DCFH-DA was used as a fluorescent probe to detect the effect of fragrant pear extract on TBHP-induced ROS in HEK-293 cells on a flow cytometer. The average fluorescence intensity directly reflects the level of intracellular ROS. The results are shown in Figure 7. Compared with the normal control group, the fluorescence level of the cells in the TBHP model group was significantly increased (*p* < 0.001), and the fluorescence level of the cells was significantly decreased after the positive drug rutin treatment (*p* < 0.001), further indicating that TBHP-induced HEK-293 The cell oxidative damage model was established successfully. Compared with the TBHP model group, after the intervention of Nanguo pear, Aksu Juju pear, Hongxiang pear and Luntai Juju pear extracts, the levels of ROS and fluorescence in HEK-293 cells were significantly decreased (*p* < 0.05). And with the ripening of fragrant pear fruit, the ability of pear extract to scavenge ROS gradually decreased. It showed that the pear extract could effectively inhibit the increase of ROS level in HEK-293 cells caused by TBHP, and reduce the oxidative damage of ROS to the cell membrane and intracellular substances.

### Cytoprotective activity of fragrant pear in different periods

**FIGURE 8:**
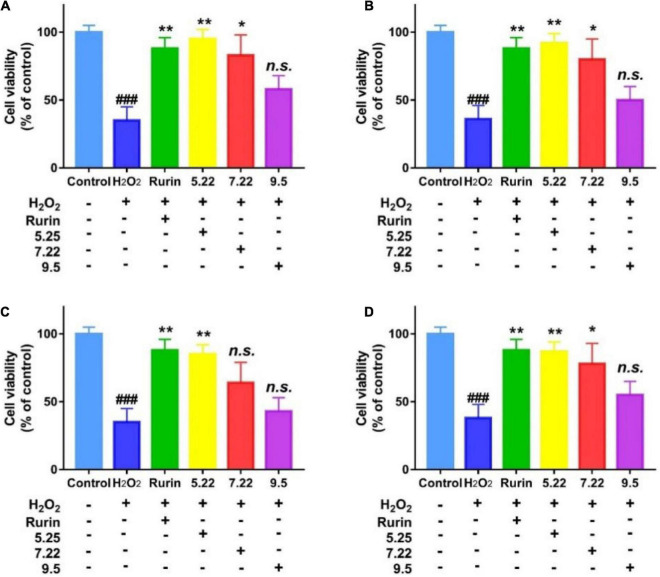
Protective effects of nanguo pear **(A)**, Aksu Juju pear **(B)**, Hongxiang pear **(C)** and Luntai Juju pear **(D)** on viability losses in HEK-293 cells stimulated by hydrogen peroxide. Cells (100 μL, 1.0 × 10^6^ CFU/mL) were treated with 200 mg/mL extracts of Nanguo pear, Aksu Juju pear, Hongxiang pear and Luntai Juju pear for 0.5 h, and then incubated with hydrogen peroxide for 6 h, and then the cell viability of HEK-293 cells were measured by MTT method. All data represent means ± SD of three independent experiments. ^###^*p* < 0.001, compared with control group, **p < 0.01 and *p < 0.05 related to H_2_O_2_ alone.

External stressor stimulation leads to oxidative damage to cells, and the degree of cell damage is directly reflected by the cell survival rate (31). In this study, H_2_O_2_ induced HEK-293 cells to establish an *in vitro* oxidative damage model, and the results are shown in Figure 8. Compared with the blank control, H_2_O_2_ significantly reduced the survival rate of HEK-293 cells (*p* < 0.05), which indicated that H_2_O_2_ induced HEK-293 cells to successfully establish an oxidative damage model. Compared with the model group, the mature fragrant pear extract (September 5) had no significant effect on the survival rate of HEK-293 cells (*p* > 0.05); picked on July 22 (except Hongxiang pear) and May 22 The fragrant pear extracts of both can significantly improve the cell survival rate (*p* < 0.05), and the fragrant pear extracts picked on May 22 had the best cytoprotective activity. The results showed that the extract of fragrant pear at the young fruit stage had a certain protective effect on HEK-293 cells, and could inhibit the oxidative damage of H_2_O_2_ to HEK-293 cells.

### Effect of different period of fragrant pear on that expression level of melanin-related protein

**FIGURE 9:**
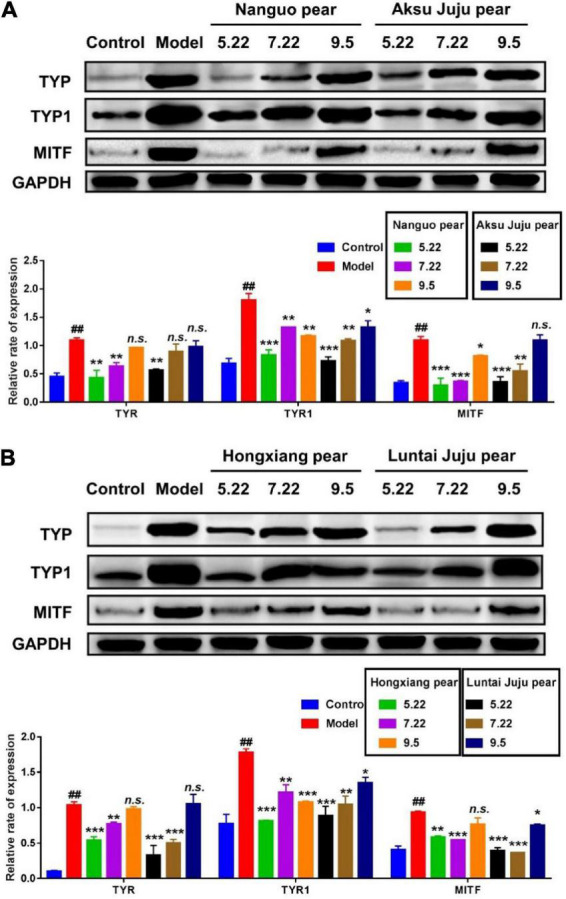
Efect of pear extracts on α-MSH induced melanin production-related protein levels in B16 cells for 48 h. Western blot analysis of TYR, TYR1, and MITF levels after Nanguo pear and Aksu Juju pear **(A)**, Hongxiang pear and Luntai Juju pear extracts **(B)** treatment. GAPDH protein was used as an internal control. All data represent means ± SD of three independent experiments. ^##^*p* < 0.01, compared with control group, ***p < 0.001, **p < 0.01 and *p < 0.05 related to model alone.

B16 cells are often used as the first choice for screening the efficacy of whitening agents, and their melanin synthesis function is basically the same as that of normal human melanocytes. The distribution and quantity of melanin in the skin determines the color of the skin, and the rate-limiting enzyme for melanin synthesis are tyrosinase-related protein (TRP-1 and TRP-2) and MITF (32). In this study, the effects of different stages of fragrant pear fruit extract on the expressions of MITF, Tyrosinase and TYRP1 in B16 cells, which are involved in tyrosine-based melanogenesis were determined by western blot experiments, and the results are shown in Figure 9. Compared with the blank control, after α-MSH induction, the expression levels of TYR, TYR1 and MITF in B16 cells were significantly increased, indicating that the α-MSH induction model was successfully established (*p* < 0.01). After treatment with fragrant pear extracts picked on May 22, the expression levels of three proteins critical for melanin production, TYR, TYR1 and MITF, were significantly decreased in B16 cells. However, with the ripening of fragrant pear fruit, the ability of fragrant pear extract to inhibit melanin production gradually decreased, especially in the mature fragrant pear fruit, which basically lost the ability to inhibit black production. As shown in the Figure 5, this result is consistent with that of arbutin content, indicating that whitening activity is significantly positively correlated with arbutin content in pear fruit. Arbutin is a natural active substance derived from green plants, which can effectively inhibit the activity of tyrosinase in the skin and block the formation of melanin (33). It could be deduced that if the whitening activity of pears was to be considered, the picking period should be selected at the young fruit stage, when the pears were rich in arbutin and had the strongest whitening activity.

## Conclusion

In this study, the dynamic change law of phenolic compounds and the antioxidant activity of fragrant pear in different periods were studied based on HPLC technology using fragrant pear in different periods as the material. The results showed that the total phenol and total flavonoids contents of the four kinds of pears were the highest in nanguo pear, the second in Akzogou pear, and the lowest in Hongxiang pear. The DPPH radical scavenging ability, ABTS^+^ radical scavenging ability and iron ion reducing ability were also the strongest in nanguo pear. And during the young fruit stage, fragrant pear had the strongest ability to remove ROS and played a very good role in cell protection. Finally, in this study, the mouse B16 cells were used as the research object, and the α-MSH-induced high-expression melanin model of B16 cells was constructed. The expressions of TYR, TYR1 and MITF proteins in B16 cells were detected by western blot. The results showed that young fragrant pear Fruit could significantly inhibit the expression of melanin-related proteins (TYR, TYR1 and MITF) in B16 cells, which provided a theoretical basis for the application of young fragrant pear Fruit as a natural whitening active substance in cosmetics or functional foods.

## Data availability statement

The original contributions presented in this study are included in the article/supplementary material, further inquiries can be directed to the corresponding authors.

## Author contributions

C-YW, H-JB, and L-RS provided research funds and designed experiments. FW collected the samples and completed the determination of phenolic compounds. HJ completed the experiment of cell protection and whitening activity. XJ performed the antioxidant experiment *in vitro*. HJ, Y-FP, and FW analyzed the data. H-JB and HJ wrote, revised, and edited this article. All authors listed have made a substantial, direct, and intellectual contribution to the work, and approved it for publication.
